# The role of phonology in lexical access in teenagers with a history of dyslexia

**DOI:** 10.1371/journal.pone.0229934

**Published:** 2020-03-17

**Authors:** Hazel I. Blythe, Jonathan H. Dickins, Colin R. Kennedy, Simon P. Liversedge

**Affiliations:** 1 Department of Psychology, Northumbria University, Newcastle, United Kingdom; 2 Psychology, University of Southampton, Southampton, United Kingdom; 3 Medicine, University of Southampton, Southampton, United Kingdom; 4 School of Psychology, University of Central Lancashire, Preston, United Kingdom; University of Oslo, NORWAY

## Abstract

We examined phonological recoding during silent sentence reading in teenagers with a history of dyslexia and their typically developing peers. Two experiments are reported in which participants’ eye movements were recorded as they read sentences containing correctly spelled words (e.g., church), pseudohomophones (e.g., *cherch*), and spelling controls (e.g., *charch*). In Experiment 1 we examined foveal processing of the target word/nonword stimuli, and in Experiment 2 we examined parafoveal pre-processing. There were four participant groups–older teenagers with a history of dyslexia, older typically developing teenagers who were matched for age, younger typically developing teenagers who were matched for reading level, and younger teenagers with a history of dyslexia. All four participant groups showed a pseudohomophone advantage, both from foveal processing and parafoveal pre-processing, indicating that teenagers with a history of dyslexia engage in phonological recoding for lexical identification during silent sentence reading in a comparable manner to their typically developing peers.

## Introduction

Our aim was to examine the role of phonological recoding during silent sentence reading in teenagers, both with and without a history of dyslexia. Phonological recoding is an effortless and subconscious process whereby a reader accesses the abstract phonological representation of a word from its orthography [1], and is considered a vital component of lexical identification in skilled adult readers [2]. There is, however, robust evidence that deficits in certain phonological awareness tasks are a key factor related to the reading difficulties associated with dyslexia [3]. It is not precisely understood how these deficits impact upon silent reading, nor how the ability to phonologically recode written words typically develops. Here, eye tracking was used to investigate differences in phonological and orthographic processing between teenagers with and without a history of dyslexia during silent sentence reading.

One of the most influential theories for the reading difficulties associated with dyslexia is the phonological deficit hypothesis [4,5,6,7,8]. This posits that children with dyslexia have fundamental difficulties with the storage and retrieval of the speech sounds associated with words, impacting on their ability to learn grapheme-phoneme correspondences, a vital aspect of reading acquisition [9] and an ability that underpins phonological recoding. Children with dyslexia perform poorly on phonological awareness tasks such as phoneme manipulation [10,11,12], and have poor verbal short term memory [3,13] as evidenced by poor performance on nonword repetition and digit span tasks. Under the phonological deficit theory, such deficits are the key causal factor leading to the reading difficulties associated with dyslexia [3,5]. Alternative theories [14,15,16] have acknowledged these deficits as a key mediator among various other neurological factors, leading to a widespread concurrence on the importance of phonological deficits in dyslexia.

In contrast, there is some evidence to suggest that phonological processing can function normally in individuals with dyslexia [17]. A semantic categorisation task was used, with both word and nonword foils, across samples of children, teenagers, and adults with dyslexia, as well as reading age- and chronological age-matched typically developing control groups. Participants with dyslexia showed the same (pseudo)homophone advantage as their typically developing peers (if anything, the magnitude of the effect was larger in the groups with dyslexia). Some researchers have, indeed, argued that individuals with dyslexia have normal cognitive representations of phonology, and that the difficulties associated with dyslexia may stem from access to those representations [18]. That said, the tasks used to demonstrate such a deficit are often somewhat artificial and require overt processing of phonology [18,19]; the extent to which such phonological awareness difficulties may occur during or impact on more natural silent reading tasks is not clear.

By using a research method such as eye tracking to investigate on-going linguistic processing during silent sentence reading, where participants are not required to pronounce letter strings, or explicitly decide upon their lexical status or semantic category, we can investigate the cognitive processes underlying reading without extraneous task demands. The ease of a reader’s cognitive processing of text is reflected in their eye movement behaviour and, accordingly, there are well-documented differences between individuals with and without dyslexia–readers with dyslexia typically make more, and longer, fixations, more regressions, and shorter saccades than typically developing readers [20,21,22]. These different patterns of eye movement behaviour from children with dyslexia are widely accepted as a consequence rather than a cause of their reading difficulties [20,21,22,23,24]. To date, however, eye movement behaviour has not been used to examine phonological and orthographic processing during lexical identification in sentence reading for individuals with dyslexia.

An important experimental question concerns whether phonological information is processed prior to or following lexical access. If the former is true, phonological information may also influence lexical access itself. Evidence for prelexical phonological processing has been provided by a variety of tasks, such as naming [25,26,27], lexical decision [28,29,30,31], and semantic categorisation [32,33]. The extent to which the extraneous demands of each task influences phonological processing, however, remains unclear [34] and the clearest evidence for prelexical phonological processing has been provided by eye tracking studies [2,35,36,37], strongly suggesting a vital role for phonological recoding in lexical access in skilled adults readers. In addition to processing during direct fixation, skilled adult readers are also able to process the phonology of upcoming words in the sentence; during fixations on word N, readers begin to process the phonology of word N+1 (parafoveal pre-processing) [37]. Interestingly, the extent to which adult readers are able to pre-process phonology seems to be dependent on reading level, with such effects only occurring in more skilled readers [35].

Note that there is a critical distinction between phonological decoding and recoding [1]. Decoding is the conscious, effortful process whereby a reader, either overtly through pronunciation or by reciting mentally, sounds out the constituent phonological units of a word. With increased experience, however, readers transition to whole word processing through phonological recoding—the rapid, pre-lexical, and covert activation of abstract phonological codes [38,39]. In these studies, children were presented with homophones or pseudohomophones—words or nonwords, that share phonology with a real word (e.g., *hear* and *here*; *cherch* and *church*)—embedded in meaningful sentence contexts. First, reading times on these (pseudo)homophones were faster than reading times on spelling controls that did not share phonology, but were matched to the (pseudo)homophone in terms of their orthographic overlap with the real word (e.g., *cherch* and *charch* both differ from *church* by the substitution of vowel, in the same location within the stimulus). This advantage to reading times for (pseudo)homophones over spelling controls reflects the reader’s beneficial processing of phonology [40]. Second, when compared against reading times on the correct target word within a sentence, the cost associated with real word homophones was less than 20ms [39] and the cost for nonword pseudohomophones was less than 200ms [38]. Furthermore, reading times on the homophones was less than 400ms, and on the pseudohomophones was less than 600ms in total. These small increases in reading times are not consistent with the possibility that participants were engaging in any overt decoding process to identify the (pseudo)homophones (e.g., subvocally sounding out the stimuli). Rather, the data strongly indicate that the (pseudo)homophone advantage observed in typically developing children as young as 7-years-old resulted from phonological recoding.

Little is known about how developing readers with dyslexia transition from decoding to recoding. In the case of a developmental delay, readers with dyslexia would, with continued reading instruction, transition to recoding at a later stage than their typically developing peers (hence our recruitment of teenage readers). Alternatively, there could be a cognitive processing deficit that fundamentally affects decoding ability and prevents the transition to phonological recoding. The present experiments were designed to allow for differentiation between these two possibilities–delayed versus atypical development of phonological recoding during reading.

Very little research has been done on this topic with this particular participant population. English teenagers with dyslexia, aged 14–16 years, were reported to make significantly more errors on a phonological judgement task than their typically developing peers when presented with pseudohomophones and nonwords, but not when asked to make an orthographic judgement [41]. This supports the argument that phonological processing difficulties persist into teenage years in individuals with dyslexia. In contrast, a study with German teenagers with dyslexia reported that teenagers with dyslexia were able to make a phonological distinction between pseudohomophones and real words, but performed poorly when asked to make an orthographic distinction [42]. They argued that, in the case of German as a regular orthography, dyslexia results in impairments to the orthographic lexicon. Thus, it seems clear that for teenagers with dyslexia, phonological processing during reading is affected by the regularity of the language. In both these studies, however, the target words and nonwords were presented in isolation. We examined phonological recoding during silent sentence reading for teenagers with dyslexia reading English, a language with an opaque orthography, both during direct fixation (Experiment 1) and during parafoveal pre-processing (Experiment 2).

## Experiment 1

Participants silently read sentences that each contained a target word/nonword whilst their eye movements were recorded. The phonological and orthographic characteristics of the target word were manipulated such that participants read a sentence containing either a correctly spelled target word (e.g., *cheese*), a pseudohomophone (e.g., *cheeze*), or a spelling control (e.g., *cheene*). The degree of orthographic overlap within each correct word/pseudohomophone/spelling control triplet was also manipulated; half of all triplets were orthographically similar (e.g., *cheese*/*cheeze*/*cheene*), with only one letter altered, and half were orthographically dissimilar (e.g., *ball/borl/bewl*), with two or more letters altered. Clearly, without meaningful context, it might be difficult to identify that a letter string such as *bewl* represented the real word *ball*. We wished to avoid highly atypical patterns of eye movement behaviour due to participants engaging in some sort of problem solving strategy when they encountered the nonwords, and so sentence frames were written to be highly semantically constraining for the target word (e.g., *Gareth threw the rugby bewl to his friend who caught it*).

With respect to overall patterns of eye movement behaviour, we predicted that that teenagers with a history of dyslexia would exhibit longer and more fixations, more regressions, shorter saccades, and longer overall reading times, relative to their typically developing peers. Such effects have been previously documented during silent sentence reading tasks with younger readers with dyslexia [20,21,22], and with German teenagers with dyslexia [43], but not in the English teenaged population. Regarding the influence of the target word manipulations upon eye movement behaviour, we predicted that typically developing teenagers would show: (a) a pseudohomophone advantage (faster reading times on the pseudohomophones relative to the spelling controls), indicative of phonological recoding; and (b) faster reading times on nonwords that were orthographically similar to their correctly spelled counterparts than on nonwords that were orthographically dissimilar. These predictions were made on the basis of data from previous studies with both skilled adult readers and younger, typically developing children [2,38,39].

With respect to investigating phonological recoding by teenagers with a history of dyslexia, there were three possible outcomes: (1) no pseudohomophone advantage (indicative of atypical development); (2) a pseudohomophone advantage in older but not younger teenagers (indicative of a developmental delay); or (3) a pseudohomophone advantage in showing that, by the time they have progressed into secondary education, individuals with a history of dyslexia have transitioned to phonological recoding.

## Method

### Participants

Participants were: (1) older teenagers with a history of dyslexia (DO); (2) younger teenagers with a history of dyslexia (DY); (3) older typically developing teenagers (TDO) matched to the DO group on chronological age; and (4) younger typically developing teenagers (TDY), matched to the DO group for word reading accuracy and to the DY group for chronological age (see Table 1). All had English as their first language, and all had normal or corrected-to-normal vision. All participants in Groups 1 and 2 had a documented history of dyslexia and had received a formal, independent diagnosis, typically by an Educational Psychologist following referral from the school, prior to being recruited for the study. This research was approved by the Ethics Committee in Psychology at the University of Southampton (submission ID 11298). All participants provided written informed consent.

**Table 1:** Participant group means for age, reading ability, phonological processing ability, nonverbal IQ, and vocabulary for Experiment 1 (standard deviations in parentheses).

		TDO	DO	TDY	DY
Experiment 1	N	30	22	26	10
	Age, in months	207 (5)	207 (14)	178 (5)	175 (8)
	Word reading, raw	125 (3)	118 (4)	120 (6)	111 (8)
	Word reading, SS	109 (6)	96 (8)	101 (10.)	88 (14)
	Pseudoword decoding, SS	103 (7)	88 (12)	99 (10)	85 (9)
	Nonverbal IQ, SS	90 (15)	93 (9)	93 (16)	91 (16)
	Vocabulary, SS	105 (7)	100 (10)	100 (9)	98 (10)
	Phonological processing, SS^a^	96 (13)	90 (10)	94 (13)	92 (17)
Experiment 2	N	30	21	21	14
	Age, in months	207 (5)	206 (11)	177 (4)	173 (10)
	Word reading, raw	125 (3)	118 (4)	120 (5)	108 (9)
	Word reading, SS	108 (6)	95 (8)	102 (10)	85 (15)
	Pseudoword decoding, SS	102 (7)	88 (12)	99 (9)	83 (10)
	Nonverbal IQ, SS	90 (15)	93 (9)	94 (17)	91 (15)
	Vocabulary, SS	104 (7)	101 (10)	100 (8)	97 (12)
	Phonological processing, SS^a^	95 (12)	90 (10)	96 (12)	90 (17)

SS denotes a standardised score.

^a^sum of subtest standardised scores. TDO = typically developing, older; DO = dyslexic, older; TDY = typically developing, younger; DY = dyslexic, younger.

All participant groups were matched on nonverbal IQ. The two older participant groups (TDO and DO) were matched on chronological age, but the group with a history of dyslexia had significantly lower scores for word reading and pseudoword decoding. The older group with a history of dyslexia (DO) were matched to the younger typically developing teenagers (TDY) on word reading, but were significantly older and had poorer pseudoword decoding accuracy. The two younger groups (TDY and DY) were matched on chronological age, but the group with a history of dyslexia had significantly lower scores for word reading and pseudoword decoding. Note that word reading scores for teenagers with a history of dyslexia were within the lower range of what is labelled "average", more so for the older group, even though they had received a formal independent diagnosis of dyslexia earlier in their education. This is likely due to the fact that we recruited the teenagers from a well-performing school in a relatively affluent area. The typically developing teenagers had reading scores that were in the upper range of "average", supporting this suggestion. As noted in the Introduction, dyslexia has been extensively studied in younger children but is somewhat less well understood in the teenage population, where individuals will often have been diagnosed at a fairly young age and then experienced a systematic programme of support and training for their reading over a number of years. Critically, both groups with a history of dyslexia performed significantly worse on the pseudoword decoding task compared to their typically developing peers, which is widely considered to be a direct and effective means of testing an individual’s phonological decoding skills [3,44,45].

### Materials

Reading ability–the word reading and pseudoword decoding subtests of the Wechsler Individual Achievement Test (WIAT-II) [46]. Phonological processing–the elision and blending words subtests of the Comprehensive Test of Phonological Processing (CTOPP) [47]. The two subtest scores were summed to give an overall score for phonological awareness. Vocabulary–the British Picture Vocabulary Scale (BPVS) [48]. Nonverbal IQ–Ravens Progressive Matrices (RPM) [49]; forty minutes is the standard assessment time for this measure, but in the present experiment all participants were given a shorter administration time in order to complete all assessments in one session. The shorter administration time means that the raw scores systematically underestimate IQ, such that standardised scores cannot be interpreted as an estimate of IQ in relation to the broader population. They remain, however, useful for the purpose of group matching, as all participants received the same administration time.

### Apparatus

An EyeLink 2K eye tracker (SR Research, Toronto, Canada) was used to record monocular eye movements from the right eye, although viewing was binocular. The position of the participant’s eye was recorded every millisecond. Sentences were presented on a 19” Viewsonic CRT monitor operating at 100Hz (120Hz for one participant) at a viewing distance of 60 cm. Sentences were presented in black, Courier New font size 14 on a grey background. Participants leaned on a chinrest and a forehead rest during the experiment to keep head movements to a minimum, and used a Microsoft gamepad to answer comprehension questions and target test questions, and to terminate each sentence.

### Stimuli and design

Twenty-four triplets of target words/nonwords were created. Each triplet was comprised of a correctly spelled word, a pseudohomophone of that target word, and a spelling control nonword, that were matched for length (all being 4–6 letters long). The spelling control nonword was created by mirroring the orthographic change between the correctly spelled word and the pseudohomophone, to create a nonword that did not share phonology with the correct target word. Pseudohomophones and spelling controls were matched on the number of syllables, consonant-vowel structure, and patterns of ascending and descending letters; in addition, we ensured that the spelling controls were always orthographically legal and pronounceable. Twelve of these triplets were classed as orthographically similar, where the nonword differed from the correct target word on just one letter, which was never the first or second letter (e.g., *church*/*cherch*/*charch*). The remaining 12 triplets were classed as orthographically dissimilar in that two or three letters were changed from the correct word, and at least one of these substitutions affected the first and/or second letter of the word (e.g., *ball/borl/bewl*). The two lists of correct target words were matched on number of orthographic neighbours (0–23 neighbours), adult frequency (0–1882 per million) [50], child frequency (8–560 per million) [51], and Age of Acquisition (150–358) [52] (all *ts* <2, all *ps* >.1). There were slight differences in word length between orthographically similar triplets (mean length = 5.33 letters) and dissimilar triplets (mean length = 4.67 letters; *t* (22) = 2.1, *p* = .05). Two sentence frames were written for each target word/nonword triplet, that were semantically constraining toward the identity of the correctly spelled target word (e.g., *My sister got married in an old stone* *church* *in Scotland* and *The vicar prayed in the old* *church* *every day even though it was cold*). These sentences were selected from a broader set that were extensively pre-screened to ensure both semantic constraint for the target word, and their suitability for use with readers of the level that we recruited for the present study. For each triplet, one sentence frame was used in Experiment 1 and the other sentence frame in Experiment 2 so that, in each experiment, each participant read 24 experimental sentences. Full details of the pre-screening, and the full list of stimuli have already been published [53]. Note that the pre-screening was conducted with 8–9 year old children, so as to be confident that the teenagers with a history of dyslexia who took part in the eye movement study would be able to read and understand the sentences despite their reading difficulties (these teenagers being recruited from Years 9–13, so possibly as young as 13 and with severe reading difficulties, depending on voluntary participation).

Each participant read the sentences in a randomised order, with the target word or nonword in each sentence rotated across three counterbalanced files. A simple comprehension question followed 25% of sentences, requiring a yes or no response using the button pad, to ensure participants were reading the sentences for meaning. It was important that participants were able to identify what the target nonwords were meant to be from the sentence context. To account for this, a ‘target test’ question was also included after roughly 50% of the trials in which participants read a pseudohomophone or control nonword. Participants were reminded that a word had been spelled incorrectly in the immediately preceding trial, and were given two options as to what the incorrectly spelt word should have been. The two options were the correct target word (e.g., *nose*) and a distractor word matched on both length and on the number of letter changes between the correct word and the target nonword in the sentence (e.g., *none*). Distractor items were also matched on child and adult frequency to the correct target words. After any given sentence, participants were only asked one of the two types of question.

### Procedure

Participants were instructed to read silently for meaning, and to expect questions relating to the sentence they had just read. A calibration procedure was completed; participants were required to fixate three stationary dots as they sequentially appeared across a horizontal array. If the mean gaze-position error was greater than 0.2°, or for any one of the three calibration points individually, the calibration procedure was repeated as necessary. On each trial, participants fixated a central point, followed by a gaze-contingent cross on the left of the screen, which triggered the appearance of the sentence when fixated. Participants read four practice sentences: two were followed by a comprehension question; two were followed by a target test. The experimental trials were then completed. After the eye movement experiment, which lasted approximately 15 minutes, each participant completed the word reading and pseudoword decoding subtests of the WIAT-II, the elision and blending words subtests of the CTOPP, and the BPVS. These assessments lasted approximately 30 minutes. Participants then completed a second eye movement experiment (see Experiment 2, approximately 15 minutes in duration), before finishing the test session by completing a 20-minute timed version of Raven’s Standard Progressive Matrices.

## Results

All data for Experiments 1 and 2 can be viewed at https://osf.io/s8j2n/?view_only=0ef9e7351d114ac3ab8e0d7fc05c4849. All participants scored at least 75% (equivalent to making a maximum of two mistakes) on the comprehension questions, and at least 90% on the target tests (equivalent to making one mistake), confirming that they were able to read and understand the sentences; there was no difference across the groups on either score (*p*s > 0.2). The data were trimmed using the clean function in the EyeLink Dataviewer software (SR Research, Ontario, Canada). In the first two stages, fixations shorter than 80ms were merged with the neighbouring fixation if within a 0.5 degree distance of another fixation over 80ms, and fixations shorter than 40ms were merged with neighbouring fixations if within a 1.25 degree distance of another fixation. In the third stage, if a target word/nonword had three or more fixations shorter than 80ms, these were merged into a single, longer fixation. Finally, all remaining fixations that were shorter than 80ms or longer than 1200ms were deleted. This process removed 2.2% of the data, resulting in a final dataset of 44,598 fixations.

The data were analysed using the lme4 package [54] within the R environment for statistical computing [55] with Linear Mixed Effects (LME) models. All models contained participants and items as random factors, with the full random structure where possible. Where models failed to converge, the random structure of each model was pruned until the model converged. All fixation duration measures were log-transformed in order to reduce distributional skewing [56]. Effects were estimated using the lmer function of the lme4 package.

### Global analyses

We examined total sentence reading times, the number of fixations per sentence, word skipping probability, and the mean number of regressions per sentence. The model syntax was dependent variable ~ group + (1|participant) + (1+group|triplet number). All models were run using the sdif function for the Group factor (for successive-differences contrast coding), with levels in the following order: (1) typically developing, older; (2) dyslexic, older; (3) typically developing, younger; and (4) dyslexic, younger (note that, in using this function, nonadjacent levels of the factor are not compared). Means and standard deviations for the dependent measures, as well as the model outputs, are reported in Table 2.

**Table 2:** Means and fixed effects estimates for Experiment 1 analyses of global measures.

	Sentence reading time			Fixation count			Skipping probability			Regression count		
	Mean (standard deviation)			Mean (standard deviation)			Mean			Mean (standard deviation)		
Typically developing, older (TDO)	3745 (1175)			15.79 (4.63)			0.41			4.05 (2.58)		
Dyslexic, older (DO)	5316 (1857)			19.87 (5.94)			0.33			4.90 (2.73)		
Typically developing, younger (TDY)	4290 (1648)			16.60 (5.30)			0.38			4.05 (2.57)		
Dyslexic, younger (DY)	6512 (3733)			23.30 (9.54)			0.29			5.63 (3.67)		
	b	SE	t	b	SE	t	b	SE	z	b	SE	t
Intercept (grand mean)	8.42	0.04	239.36	18.90	0.60	31.30	-0.64	0.05	13.04	4.66	0.25	18.44
Group (TDO vs. DO)	0.34	0.07	5.07	4.08	1.07	3.82	-0.37	0.09	4.10	0.85	0.50	1.70
Group (DO vs. TDY)	-0.23	0.07	-3.28	-3.27	1.11	-2.96	0.25	0.09	2.76	-0.85	0.52	-1.64
Group (TDY vs. DY)	0.38	0.09	4.21	6.72	1.47	4.57	-0.45	0.12	3.71	1.58	0.67	2.34

Sentence reading times are in ms. Fixation and regression counts were calculated per sentence. Skipping probability was calculated across all participants in each group, and across all words in the sentences. Note that *b* is the beta-estimate for effect size (for reading time measures, based on log-transformed values); *SE* is the standard error, *t* is the t-value, and *z* is the z-value for each term. T-values approximate the z-score distribution, and where *t* = 1.96 then *p* = 0.05. Here, we adopt a significance criterion of *t* > 2 for significance.

For total sentence reading time, we observed differences across all three key group comparisons–reading times were longer for the older teenagers with a history of dyslexia than either their chronological age- or word reading accuracy-matched typically developing peers, and the younger teenagers with a history of dyslexia also showed longer reading times than their chronological age-matched typically developing peers. We observed the same pattern of differences for the number of fixations per sentence, such that teenagers with a history of dyslexia made more fixations per sentence than the typically developing teenagers across all three comparisons, and word skipping probability, such that the teenagers with a history of dyslexia were less likely to skip words in the sentences than the typically developing teenagers. Finally, we observed a consistent pattern in the number of regressions per sentence, although not all three comparisons were statistically reliable. Older teenagers with a history of dyslexia showed a non-significant trend for more regressions per sentence compared to the older and younger typically developing teenagers. The younger teenagers with a history of dyslexia made significantly more regressions per sentence than their typically developing, chronological age-matched peers. In summary, these analyses clearly demonstrate greater processing difficulty during reading for teenagers with a history of dyslexia when compared to both chronological age-matched and word reading accuracy-matched typically developing teenagers.

### Local analyses

We report analyses of single fixation duration (fixation duration in cases where only one first pass fixation was made on the target word/nonword), first fixation duration (the duration of the initial first pass fixation on a target word/nonword, regardless of whether or not that word received subsequent refixations), and gaze duration (the sum of all first pass fixations on the target word/nonword, before the eyes moved to a different word within the sentence).

The unbalanced design of this experiment warranted a two step approach to the local analyses. There was no meaningful comparison to be made between the two lists of correctly spelled target words based on which orthographic similarity category they were in, as orthographic similarity referred to the relationship between a correct target and its two nonword partners within each triplet. For Model 1, therefore, data from all correctly spelled words were collapsed into a single condition, and these reading times were compared to those from each of the four nonword conditions. In Model 2, data from correctly spelled words were excluded from the analysis, and we examined the effects of our manipulations of phonological and orthographic overlap within the four nonword conditions only.

### Model 1

Means and standard deviations for local measures of eye movement behaviour between groups can be observed in Table 3, and fixed effects estimates are shown in Table 4. The correctly spelled target words (e.g., *church*/*ball*), were compared with each of the four types of misspelled words that participants were presented with: 1) Orthographically similar pseudohomophones (e.g., *cherch*); 2) Orthographically dissimilar pseudohomophones (e.g., *borl*); 3) Orthographically similar spelling controls (e.g., *charch*); 4) Orthographically dissimilar spelling controls (e.g., *bewl*). An LME model was constructed with group and target type as fixed factors with an interaction between the two (dependent variable ~ group*target type +(1+target type|participant) + (1+group*target type|triplet number).

**Table 3:** Means for local eye movement measures for Experiment 1.

	TDO		DO		TDY		DY	
	Orth. similar	Orth. dissimilar	Orth. similar	Orth. dissimilar	Orth. similar	Orth. dissimilar	Orth. similar	Orth. dissimilar
Single fixation duration								
Correct targets	212 (92)		219 (63)		221 (76)		253 (121)	
Pseudohomophones	226 (98)	282 (141)	259 (157)	326 (190)	266 (145)	285 (149)	237 (81)	314 (127)
Spelling Controls	264 (109)	285 (162)	244 (91)	362 (213)	287 (147)	313 (178)	338 (141)	329 (239)
First fixation duration								
Correct targets	208 (86)		221 (77)		221 (74)		250 (115)	
Pseudohomophones	224 (91)	270 (128)	262 (143)	298 (163)	255 (130)	280 (146)	239 (85)	290 (119)
Spelling Controls	255 (115)	280 (149)	281 (150)	343 (197)	283 (131)	303 (162)	320 (164)	282 (188)
Gaze duration								
Correct targets	244 (128)		275 (148)		245 (120)		322 (179)	
Pseudohomophones	318 (177)	349 (178)	378 (266)	469 (289)	358 (230)	382 (227)	477 (367)	483 (276)
Spelling Controls	368 (182)	417 (292)	454 (312)	568 (402)	396 (235)	478 (303)	489 (324)	661 (544)

All measures are reported in milliseconds. Standard deviations are in parentheses. TDO = typically developing, older; DO = dyslexic, older; TDY = typically developing, younger; DY = dyslexic, younger.

**Table 4:** Fixed effects estimates for Experiment 1 Model 1 analyses of local measures.

		Single fixation duration			First fixation duration			Gaze duration		
		b	SE	t	b	SE	t	b	SE	t
Intercept (TDO, correctly spelled)		4.54	0.07	65.21	4.50	0.06	72.12	5.09	0.06	83.05
1	Target (OS P)	0.13	0.10	1.34	0.13	0.08	1.60	0.29	0.09	3.27
2	Target (OD P)	0.38	0.10	3.82	0.38	0.08	4.72	0.39	0.09	4.45
3	Target (OS S)	0.40	0.10	3.96	0.32	0.08	3.98	0.50	0.09	5.75
4	Target (OD S)	0.35	0.10	3.47	0.40	0.08	4.93	0.44	0.09	5.06
5	Group (DO)	0.18	0.10	1.86	0.21	0.09	2.33	0.20	0.09	2.28
6	Group (TDY)	0.13	0.09	1.40	0.17	0.09	1.96	0.06	0.08	0.78
7	Group (DY)	0.24	0.13	1.91	0.27	0.11	2.36	0.30	0.11	2.80
8	Group x Target (DO, OS P)	-0.06	0.15	0.44	0.00	0.12	0.01	-0.16	0.13	1.23
9	Group x Target (DO, OD P)	-0.12	0.15	0.78	-0.16	0.12	1.32	-0.09	0.13	0.73
10	Group x Target (DO, OS S)	-0.33	0.15	2.21	-0.12	0.12	0.97	-0.34	0.13	2.65
11	Group x Target (DO, OD S)	-0.03	0.15	0.21	-0.03	0.12	0.22	-0.27	0.13	2.10
12	Group x Target (TDY, OS P)	0.01	0.14	0.10	-0.04	0.12	0.35	-0.07	0.12	0.55
13	Group x Target (TDY, OD P)	-0.11	0.14	0.82	-0.13	0.11	1.09	-0.05	0.12	0.44
14	Group x Target (TDY, OS S)	-0.07	0.14	0.48	0.02	0.12	0.18	-0.19	0.12	1.55
15	Group x Target (TDY, OD S)	0.01	0.14	0.05	-0.06	0.12	0.54	-0.05	0.12	0.38
16	Group x Target (DY, OS P)	-0.10	0.21	0.45	-0.11	0.15	0.74	-0.11	0.16	0.66
17	Group x Target (DY, OD P)	-0.09	0.19	0.46	-0.15	0.15	0.98	-0.37	0.16	2.32
18	Group x Target (DY, OS S)	0.07	0.19	0.38	0.07	0.15	0.46	-0.33	0.16	2.07
19	Group x Target (DY, OD S)	-0.19	0.20	0.93	-0.42	0.15	2.80	-0.55	0.16	3.41

TDO = typically developing, older; DO = dyslexic, older; TDY = typically developing, younger; DY = dyslexic, younger. OS = orthographically similar; OD = orthographically dissimilar; P = pseudohomophone; S = spelling control. Note that *b* is the beta-estimate for effect size (for reading time measures, based on log-transformed values); *SE* is the standard error, and *t* is the t-value for each term. T-values approximate the z-score distribution, and where *t* = 1.96 then *p* = 0.05. Here, we adopt a significance criterion of *t* > 2 for significance.

We focus here upon the cost associated with processing nonwords, relative to correctly spelled words, and whether that cost varied across the four participant groups. Across all dependent measures, a consistent pattern emerged concerning the effect of target type. Words in all the misspelled conditions received longer reading times than correctly spelled words, with one exception; there were no significant differences in single or first fixation duration between correctly spelled words and orthographically similar pseudohomophones (Term 1 in Table 4). These effects clearly demonstrate the processing cost associated with misspelled words, although this cost was reduced for the orthographically similar pseudohomophones–nonwords that shared phonology with, and had a minimal orthographic difference from, the correctly spelled target word. There were also some significant interactions between group and target type. Relative to the older typically developing group, both older (single fixation duration and gaze duration) and younger (first fixation duration and gaze duration) teenagers with a history of dyslexia showed evidence of greater disruption from the spelling controls (Terms 10–11 and 18–19 respectively in Table 4). For example, as can be seen from Table 3, the mean cost to gaze duration associated with spelling controls relative to correctly spelled words was 149ms for the TDO group, 235ms for the DO group, and 251ms for the DY group. We did not predict this cost associated with the spelling controls for the participants with a history of dyslexia. Such an effect is likely to be associated with processing of the nonwords’ orthography and so will be explored further in Model 2, in which the effect of the orthographic manipulation is directly examined.

### Model 2

Correctly spelled target words were excluded from Model 2, in which the orthogonal manipulations of phonological (pseudohomophones vs. spelling controls) and orthographic similarity (orthographically similar vs. dissimilar) were examined across the four participant groups. These three variables were entered as interacting factors into LME models, with participant and target triplet as random factors. The syntax for the model with the full random structure was dependent variable ~ phoncond*orthcond*group + (1+phoncond*orthcond|participant) + (1+phoncond*group|triplet number). The sdif function was used to compare conditions successively, rather than iteratively to a single intercept condition. Levels of the group factor were ordered as per the global analyses. Both the phonological and orthographic manipulations only had two levels.

LME models were run for the same three dependent eye movement variables; single fixation duration, first fixation duration, and gaze duration. Fixed effects estimates for these measures can be seen in Table 5.

**Table 5:** Fixed effect estimates for Experiment 1 Model 2 analyses of local measures.

		Single fixation duration			First fixation duration			Gaze duration		
		b	SE	t	b	SE	t	b	SE	t
Intercept (grand mean)		5.56	0.03	212.99	5.53	0.02	257.37	5.88	0.04	134.28
1	Group (TDO vs. DO)	0.09	0.06	1.41	0.12	0.05	2.22	0.20	0.07	2.95
2	Group (DO vs. TDY)	-0.02	0.06	0.25	-0.03	0.05	0.66	-0.10	0.07	1.50
3	Group (TDY vs. DY)	0.06	0.08	0.74	0.01	0.06	0.08	0.22	0.09	2.34
4	Phon	0.08	0.04	1.86	0.08	0.03	2.73	0.14	0.05	2.69
5	Orth	0.11	0.04	2.81	0.08	0.03	2.44	0.14	0.08	1.85
6	Group x Phon (TDO vs. DO)	-0.03	0.08	0.39	0.02	0.06	0.39	0.03	0.08	0.31
7	Group x Phon (DO vs. TDY)	0.03	0.09	0.32	-0.01	0.06	0.17	-0.02	0.08	0.24
8	Group x Phon (TDY vs. DY)	0.06	0.12	0.55	0.00	0.08	0.01	-0.02	0.10	0.19
9	Group x Orth (TDO vs. DO)	0.16	0.10	1.66	0.01	0.08	0.14	0.14	0.08	1.67
10	Group x Orth (DO vs. TDY)	-0.21	0.09	2.37	-0.08	0.07	1.06	-0.12	0.09	1.35
11	Group x Orth (TDY vs. DY)	-0.01	0.11	0.11	-0.06	0.09	0.71	0.02	0.13	0.12
12	Phon x Orth	-0.10	0.08	1.21	-0.11	0.06	1.96	0.00	0.10	0.01
13	Group x Phon x Orth (TDO vs. DO)	0.24	0.15	1.55	0.15	0.12	1.26	0.05	0.16	0.30
14	Group x Phon x Orth (DO vs. TDY)	-0.09	0.15	0.59	-0.10	0.12	0.83	0.07	0.16	0.47
15	Group x Phon x Orth (TDY vs. DY)	-0.27	0.21	1.28	-0.28	0.15	1.88	-0.02	0.20	0.09

**TDO = typically developing, older; DO = dyslexic, older; TDY = typically developing, younger; DY = dyslexic, younger.** Note that *b* is the beta-estimate for effect size (for reading time measures, based on log-transformed values); *SE* is the standard error, and *t* is the t-value for each term. T-values approximate the z-score distribution, and where *t* = 1.96 then *p* = 0.05. Here, we adopt a significance criterion of *t* > 2 for significance.

There were overall differences between the participant groups. There was an increase in reading times for teenagers with a history of dyslexia relative to their typically developing peers for both the older groups (first fixation duration = 39ms, gaze duration = 104ms; Term 1 in Table 5) and the younger groups (gaze duration = 125ms; Term 3 in Table 5). There were no overall differences in reading times between the older group with a history of dyslexia and their word reading accuracy-matched controls (younger typically developing teenagers; Term 2 in Table 5).

**Fig 1:**
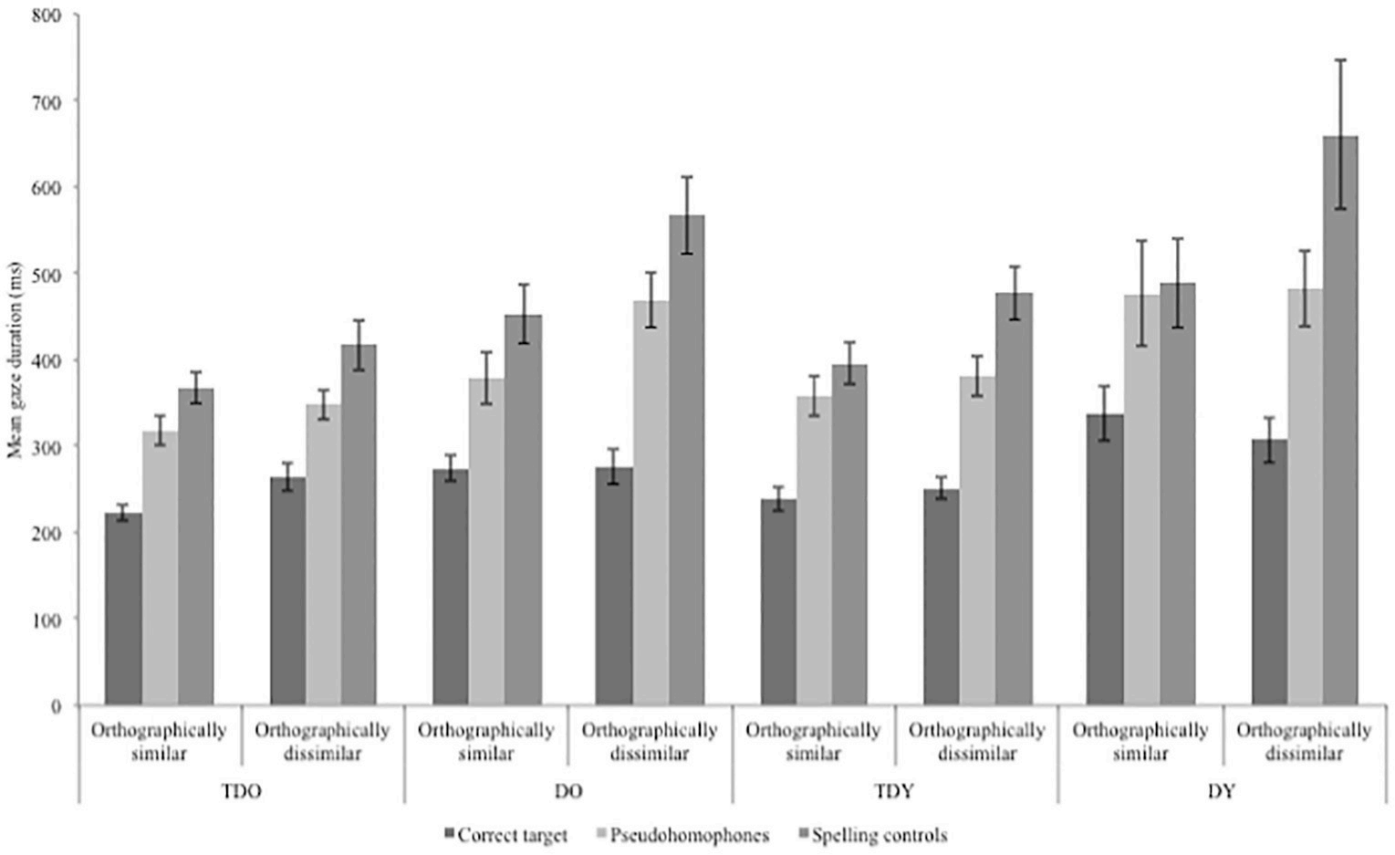
The pseudohomophone advantage in gaze duration for each of the four participant groups. TDO = typically developing, older; DO = dyslexic, older; TDY = typically developing, younger; DY = dyslexic, younger. Error bars show the standard error per group, per condition.

With respect to the manipulation of phonology, as expected, we observed overall faster reading times on pseudohomophones relative to spelling controls–a pseudohomophone advantage (single fixation duration = 23ms, first fixation duration = 26ms, gaze duration = 72ms; Term 4 in Table 5). Somewhat surprisingly, there were no interactions between participant group and phonological condition; all four groups showed a pseudohomophone advantage in the reading times (Terms 6–8 and 13–15 in Table 5; see Fig 1). We also observed an effect of orthographic similarity, such that nonwords that were orthographically similar to their correctly spelled base words received shorter reading times than nonwords that were orthographically dissimilar (single fixation duration = 44ms, first fixation duration = 31ms, gaze duration = 65ms; Term 5 in Table 5). There was one significant interaction between participant group (concerning the comparison of the older teenagers with a history of dyslexia and their younger, word reading accuracy-matched controls) and orthographic similarity condition, but this only occurred in single fixation duration. The probability of making a single fixation on the target word/nonword was 0.61 for the older teenagers with a history of dyslexia and 0.68 for the younger typically developing teenagers. Thus, although the effect did not occur across all dependent measures, these effects stem from reading times on over 50% of trials. In these cases where a single fixation was made on the target nonword, the older group of teenagers with a history of dyslexia showed a much greater cost to processing for orthographically dissimilar items (92 ms) relative to their word reading accuracy-matched controls (22 ms) (Term 10 in Table 5).

The non-significant interactions between participant group and the phonological manipulation was unexpected. With sample sizes between 10 and 30 per participant group, these analyses may have had low statistical power for detecting small effects. To examine this further, therefore, we calculated Bayes Factor for a comparison of a model where group was included as an interaction with the phonological manipulation (group*phoncond) against a denominator model without the interaction (group+phoncond; the random structure was the same for both) [57,58]. This was done using the Bayes Factor package in R [59], with 100,000 Monte Carlo iterations and with *g*-priors scaled to *r* = 0.5 for fixed effects. The Bayes factor for the original model, when compared to the denominator model was 0.01, providing strong support for the denominator model (where the group factor did not interact with the phonological manipulation). Together, our statistical analyses strongly indicate that all four participant groups showed a pseudohomophone advantage of the same magnitude and time course.

## Discussion

First, we compared overall sentence reading behaviours between our four participant groups. Differences were observed in total sentence reading time, the number of fixations per sentence, word skipping probability, and, to a lesser degree, the number of regressions per sentence, across both chronological age-matched comparisons and the reading level-matched comparison. As the first study investigating eye movements during reading in dyslexia to use both chronological age-matched and word reading accuracy-matched comparisons, these results indicate that the reading difficulties associated with a history of dyslexia stem from atypical (as opposed to simply delayed development of) cognitive processing during reading. This finding is in line with previous theories of dyslexia which suggest a fundamental deficit, rather than a developmental delay, in dyslexia [4,5,6,7,8].

We then went on to examine reading times on the target word/nonword within each sentence, specifically examining the effects of our manipulations of phonology and orthography. Teenagers with and without a history of dyslexia clearly showed a pseudohomophone advantage during silent sentence reading. There was no evidence to suggest that the pseudohomophone advantage was either absent, reduced in magnitude, or delayed for the teenagers with a history of dyslexia relative to their typically developing peers. Taking all the results together, whilst there were clear differences between the teenagers with and without a history of dyslexia in terms of their overall sentence processing difficulty, these global reading difficulties do not seem to be the result of absent or atypical phonological recoding during lexical identification when reading sentences silently for meaning. The demonstration of a robust pseudohomophone advantage in the groups of teenagers with a history of dyslexia was somewhat surprising, given the well-documented difficulties in phonological awareness that are associated with dyslexia. As discussed, prior research has demonstrated that young typically developing readers aged from 7 onwards already appeared to be sophisticated with regards to their phonological recoding during silent reading [38,39]. Little, however, is known about how this transition from decoding to recoding may occur in readers with dyslexia.

These data strongly suggest that the teenagers with a history of dyslexia were not decoding the nonword stimuli but were instead engaging in phonological recoding. If it were the case that the readers with a history of dyslexia were processing the pseudohomophones through an effortful decoding process, then the Model 1 analyses ought to have clearly shown substantially increased reading times for the pseudohomophones (relative to the correctly spelled words) for readers with a history of dyslexia. In contrast, our analyses showed that the cost associated with processing a pseudohomophone, relative to the correctly spelled target word, was no greater for readers with a history of dyslexia than for their typically developing peers. On this basis, it seems that all participants were able to process the pseudohomophones using covert phonological recoding.

The finding that teenagers with a history of dyslexia do not differ significantly to their typically developing peers with regards to phonological recoding during silent reading must be considered in relation to results from the pen and paper assessments. First, note that there were no significant differences between typically developing teenagers and teenagers with a history of dyslexia in phonological processing ability as measured by the CTOPP, a surprising finding given the phonological processing difficulties usually associated with dyslexia. Scores on the CTOPP assessment were, however, consistent with the eye movement data. Taken together, these two sets of data indicate that the teenagers with a history of dyslexia who took part in this study had good phonological processing skills, and were able to use those skills during sentence reading to engage in normal phonological recoding for lexical identification. In contrast, however, group differences were observed across all three group comparisons in pseudoword decoding, the ability to correctly pronounce an unfamiliar nonword (WIAT-II). Thus, when compared to typically developing readers at either an equivalent age or word reading accuracy, the teenagers with a history of dyslexia were impaired in their ability to phonologically decode nonword items. This discrepancy in performance between the pseudoword decoding task and the eye movement data may be related to several aspects of the tasks themselves. First, when teenagers with a history of dyslexia read a pseudohomophone during the sentence reading task, there was a lexical entry that each pseudohomophone corresponded to. It may be the case that top down activation from this lexical entry aided phonological recoding of the pseudohomophone, in a way that was not possible in the pseudoword decoding task, where the nonwords do not correspond to a lexical entry. Second, sentence context may have aided identification in the sentence reading task. Some pseudohomophones and spelling controls, particularly in the orthographically dissimilar condition, bore little resemblance to the lexical entry with which they corresponded (e.g., *honey*, *hunni*, *henma*). For this reason, each sentence was written to be semantically constraining for the target word (at least 60% of 8–9 year old children in the pre-screening study predicted each target word from its surrounding sentence context [53]). It may be that these highly constraining sentences facilitated the readers’ phonological recoding of the target words/nonwords. For example, previous work has shown that individuals with dyslexia may make greater use of sentence context to predict the identity of a word [60]. If this were the case, then the pseudohomophone advantage in the teenagers with a history of dyslexia might result from top-down processing of phonology, as the reader predicts the upcoming word and semantic, phonological, and orthographic information becomes activated prior to direct fixation. This would be quite distinct from the bottom-up phonological recoding that is thought to be required for pseudohomophones (because there are no lexical entries corresponding to pseudohomophones).

In addition to the effects of our phonological manipulation, there was an effect of orthographic similarity such that nonwords with greater orthographic overlap with their correctly spelled base word received shorter reading times than nonwords with a less orthographic overlap. This was consistent with previous studies that have examined the behaviour of skilled adult readers [2]. Interestingly, there was evidence to suggest that this effect was more pronounced in the teenagers with a history of dyslexia; specifically, the older teenagers with a history of dyslexia were particularly disrupted by orthographically dissimilar nonwords. We return to this point in the General Discussion.

In conclusion, the data from Experiment 1 provide no evidence for differential phonological recoding during sentence reading in teenagers with a history of dyslexia compared to their typically developing peers. These results clearly indicate that teenagers with a history of dyslexia are able to access abstract phonological representations during silent sentence reading, despite the overt phonological processing deficits widely associated with dyslexia. It is well known, however, that readers do not simply process information from the word that they are currently fixating. Instead, during a fixation on a word within a sentence, readers begin to extract information from the next word in the sentence (N+1)–parafoveal pre-processing. Such pre-processing is a hallmark of skilled reading, and denying the opportunity for parafoveal pre-processing is detrimental to cognitive processing during reading [61]. A substantial body of research has shown that skilled adult readers extract information about characteristics of word N+1 such as its length, orthography, and phonology [62]. Relatively little is known, however, about parafoveal pre-processing in either typically or atypically developing populations of readers [63–66]. In Experiment Two, we examined parafoveal pre-processing of phonology and orthography in teenagers with and without a history of dyslexia.

## Experiment 2

In Experiment 2, we compared parafoveal pre-processing of both phonology and orthography in teenagers with and without a history of dyslexia, who were a subset of the sample from Experiment 1. We made three predictions for Experiment 2. First, that the older, typically developing teenagers would show sensitivity to both orthography and phonology in parafovea. Our pen and paper assessments indicated that these teenagers were skilled readers (indeed, all were in higher education at the time of testing), and so such effects should be predicted on the basis of previous research [35,37,67–70]. Second, we predicted that both the older and the younger teenagers with a history of dyslexia should show sensitivity to orthography in the parafovea. These teenagers had a range of reading levels that were below what would be expected for their age, with their reading ages estimated to be 8–13 years from the word reading subtest. Parafoveal pre-processing of orthography has been shown to occur in children as young as 8-years [65]. Thus, whilst clearly exhibiting reading difficulties consistent with their diagnosis of dyslexia, even the younger teenagers with a history of dyslexia had achieved a reading level at which parafoveal pre-processing of orthography might be expected. Third, we predicted interactions between participant group (with vs. without a history of dyslexia) and the manipulation of phonology, such that there should be no pseudohomophone advantage from parafoveal preview in either of the two groups of teenagers with a history of dyslexia. Given that typically developing adult readers who have a lower level of reading skill have not been found to extract phonological information in parafoveal preview [35], then it seems highly unlikely that teenagers with a history of dyslexia will be able to.

## Method

### Participants

The same four matched participant groups were recruited as reported for Experiment 1. Sample sizes were slightly smaller, as not all individuals completed the entire experimental session. A summary of the pen and paper assessments is shown in Table 1.

### Apparatus

As in Experiment 1.

### Stimuli and design

As in Experiment 1 (note that the second, distinct sentence frame was used here for each triplet). Here, the target words/ nonwords were presented using the boundary paradigm [71]. An invisible boundary was programmed immediately after the last letter of the pre-target word. Prior to the eyes crossing that boundary for the first time, the correct words/ pseudohomophones/ spelling controls were presented in the target location. When the reader’s eyes first moved across the boundary then a display change was triggered such that the preview stimulus in the target location was replaced on all trials with the correctly spelled word. No target test questions were presented, because participants never directly fixated a misspelled word.

### Procedure

As in Experiment 1.

## Results

The data were trimmed for outliers using DataViewer software, as in Experiment 1; this resulted in the removal of 2.3% of the data, with a trimmed dataset of 39,116 fixations (outlier removal was fairly even across the four groups: DO = 3.3%; DY = 1.8%; TDO = 2.4%; TDY = 1.4%). Subsequently, trials in which the display change had occurred too early (during a fixation on any word preceding the target word in the sentence) or too late (more than 10 ms after the eyes had crossed the invisible boundary) were excluded from the analysis (18% of trials). In addition, trials in which participants skipped the pre-target word, thus having not had the opportunity for parafoveal pre-processing, were excluded from analysis (22% of trials). The relatively high proportion of excluded trials is likely due to the teenagers reading relatively simple sentences (likely resulting in larger saccade amplitudes and a higher probability of word skipping). The final data set for analysis was comprised of 1,253 trials with valid first pass reading time data for the target word (61% of the full data set). Means and standard deviations for reading times on the target word, following the display change, are shown in Table 6 as a function of preview type. Data analyses were conducted using LME models, as per Experiment 1.

**Table 6:** Means for local eye movement measures for Experiment 2.

	TDO		DO		TDY		DY	
	Orth. similar	Orth. dissimilar	Orth. similar	Orth. dissimilar	Orth. similar	Orth. dissimilar	Orth. similar	Orth. dissimilar
Single fixation duration								
Correct targets	210 (83)		225 (81)		220 (59)		271 (109)	
Pseudohomophones	223 (62)	261 (87)	264 (85)	285 (98)	238 (73)	270 (84)	305 (168)	360 (99)
Spelling Controls	243 (69)	253 (71)	253 (105)	342 (169)	252 (72)	288 (87)	317 (106)	393 (214)
First fixation duration								
Correct targets	209 (80)		227 (87)		225 (65)		272 (104)	
Pseudohomophones	214 (63)	253 (85)	265 (105)	265 (97)	237 (72)	265 (82)	276 (140)	331 (107)
Spelling Controls	235 (71)	245 (71)	245 (97)	316 (163)	252 (71)	268 (85)	307 (101)	358 (206)
Gaze duration								
Correct targets	226 (94)		254 (129)		236 (85)		331 (164)	
Pseudohomophones	234 (66)	273 (90)	291 (129)	312 (106)	240 (72)	284 (92)	367 (166)	448 (323)
Spelling Controls	267 (119)	267 (89)	281 (113)	368 (182)	259 (81)	345 (184)	402 (210)	416 (202)

All measures are reported in milliseconds. TDO = typically developing, older; DO = dyslexic, older; TDY = typically developing, younger; DY = dyslexic, younger.

### Model 1

Fixed effects estimates for Model 1 are shown in Table 7. There are three key findings from this initial analysis. First, as might be expected, in most cases a nonword preview resulted in longer reading times on the subsequently presented target word than was in the case of the identity preview (Terms 2–4 in Table 7). Second, interestingly, previews of pseudohomophones that were orthographically similar to their correctly spelled base words did not result in any cost to reading times on the target, relative to the identity preview condition (Term 1 in Table 7). Third, and again, interestingly, none of the interactions between participant groups and our experimental manipulations were statistically significant (Terms 8–19 in Table 7).

**Table 7:** Fixed effects estimates for Experiment 2 Model 1 analyses of local measures.

		Single fixation duration			First fixation duration			Gaze duration		
		b	SE	t	b	SE	t	b	SE	t
Intercept (TDO, correctly spelled)		5.25	0.05	112.24	5.26	0.04	124.20	5.31	0.05	98.78
1	Target (OS P)	0.09	0.05	1.73	0.05	0.05	0.86	0.10	0.06	1.72
2	Target (OD P)	0.24	0.06	3.95	0.21	0.06	3.32	0.22	0.06	3.39
3	Target (OS S)	0.18	0.06	2.94	0.14	0.06	2.23	0.21	0.07	3.18
4	Target (OD S)	0.25	0.05	4.81	0.20	0.05	3.73	0.24	0.06	4.24
5	Group (DO)	0.12	0.06	1.96	0.10	0.06	1.70	0.12	0.07	1.70
6	Group (TDY)	0.10	0.06	1.57	0.11	0.06	1.89	0.09	0.07	1.20
7	Group (DY)	0.32	0.07	4.60	0.28	0.06	4.46	0.39	0.08	4.89
8	Group x Target (DO, OS P)	0.05	0.08	0.63	0.09	0.08	1.19	0.05	0.08	0.68
9	Group x Target (DO, OD P)	-0.03	0.09	0.38	-0.06	0.09	0.67	0.02	0.09	0.18
10	Group x Target (DO, OS S)	-0.13	0.09	1.47	-0.07	0.08	0.89	-0.09	0.09	1.06
11	Group x Target (DO, OD S)	0.11	0.08	1.43	0.07	0.08	0.89	0.11	0.08	1.32
12	Group x Target (TDY, OS P)	-0.03	0.08	0.34	0.00	0.08	0.05	-0.06	0.09	0.71
13	Group x Target (TDY, OD P)	-0.01	0.09	0.14	-0.04	0.09	0.46	0.00	0.09	0.04
14	Group x Target (TDY, OS S)	-0.05	0.09	0.53	-0.03	0.09	0.28	-0.09	0.10	0.90
15	Group x Target (TDY, OD S)	0.04	0.08	0.46	-0.04	0.08	0.43	0.09	0.09	1.05
16	Group x Target (DY, OS P)	-0.02	0.10	0.21	-0.08	0.09	0.91	-0.03	0.09	0.27
17	Group x Target (DY, OD P)	-0.02	0.10	0.18	-0.02	0.10	0.20	0.01	0.10	0.06
18	Group x Target (DY, OS S)	-0.03	0.10	0.32	-0.02	0.09	0.21	-0.03	0.10	0.28
19	Group x Target (DY, OD S)	0.03	0.09	0.35	0.00	0.09	0.01	-0.04	0.09	0.49

TDO = typically developing, older; DO = dyslexic, older; TDY = typically developing, younger; DY = dyslexic, younger. OS = orthographically similar; OD = orthographically dissimilar; P = pseudohomophone; S = spelling control. Note that *b* is the beta-estimate for effect size (for reading time measures, based on log-transformed values); *SE* is the standard error, and *t* is the t-value for each term. T-values approximate the z-score distribution, and where *t* = 1.96 then *p* = 0.05. Here, we adopt a significance criterion of *t* > 2 for significance.

### Model 2

Fixed effects estimates for Model 2 are shown in Table 8. The pattern of results was strikingly clear. Teenagers with a history of dyslexia had longer reading times than their chronological age-matched typically developing peers, but did not differ from their word reading accuracy-matched typically developing peers (see Terms 1–3 in Table 8), replicating the overall pattern of group differences reported in Experiment 1. For example, gaze durations were 49ms longer for the DO group than the TDO group and 126ms longer for the DY group than the TDY group; in contrast, the 24ms difference between the DO and TDY groups was not significant (*t* = 1.03).

**Table 8:** Fixed effects estimates for Experiment 2 Model 2 analyses of local measures.

		Single fixation duration			First fixation duration			Gaze duration		
		b	SE	t	b	SE	t	b	SE	t
Intercept (grand mean)		5.57	0.03	160.22	5.52	0.03	199.06	5.64	0.04	154.39
1	Group (TDO vs. DO)	0.12	0.05	2.27	0.10	0.05	2.00	0.13	0.06	2.20
2	Group (DO vs. TDY)	-0.04	0.06	0.64	-0.01	0.05	0.27	-0.07	0.06	1.03
3	Group (TDY vs. DY)	0.22	0.07	3.09	0.16	0.06	2.70	0.29	0.07	4.00
4	Phon	0.06	0.04	1.66	0.06	0.03	1.68	0.07	0.03	2.13
5	Orth	0.16	0.06	2.65	0.12	0.05	2.59	0.16	0.06	2.57
6	Group x Phon (TDO vs. DO)	-0.01	0.06	0.16	-0.02	0.06	0.28	-0.02	0.07	0.29
7	Group x Phon (DO vs. TDY)	0.03	0.07	0.38	0.01	0.07	0.14	0.06	0.07	0.80
8	Group x Phon (TDY vs. DY)	-0.02	0.08	0.21	0.03	0.07	0.45	-0.08	0.08	1.01
9	Group x Orth (TDO vs. DO)	0.06	0.06	0.95	-0.01	0.07	0.13	0.09	0.07	1.31
10	Group x Orth (DO vs. TDY)	-0.02	0.07	0.29	-0.02	0.07	0.27	0.03	0.07	0.44
11	Group x Orth (TDY vs. DY)	-0.02	0.10	0.18	0.04	0.08	0.58	-0.13	0.09	1.43
12	Phon x Orth	0.01	0.07	0.21	-0.03	0.07	0.40	-0.03	0.06	0.47
13	Group x Phon x Orth (TDO vs. DO)	0.30	0.12	2.56	0.30	0.12	2.46	0.23	0.12	1.90
14	Group x Phon x Orth (DO vs. TDY)	-0.26	0.13	2.03	-0.27	0.13	2.01	-0.11	0.13	0.86
15	Group x Phon x Orth (TDY vs. DY)	0.01	0.15	0.05	-0.06	0.15	0.42	-0.17	0.14	1.21

TDO = typically developing, older; DO = dyslexic, older; TDY = typically developing, younger; DY = dyslexic, younger. Note that *b* is the beta-estimate for effect size (for reading time measures, based on log-transformed values); *SE* is the standard error, and *t* is the t-value for each term. T-values approximate the z-score distribution, and where *t* = 1.96 then *p* = 0.05. Here, we adopt a significance criterion of *t* > 2 for significance.

There was an overall pseudohomophone advantage, marginally significant in first fixation duration, and significant in gaze duration, showing that reading times on the target word were faster following a pseudohomophone preview than a spelling control preview (Term 4 in Table 8). Surprisingly, there were no significant interactions between the phonology manipulation (pseudohomophones vs. spelling controls) and any of the participant groups (Terms 6–8 in Table 8), indicating that all four participant groups showed a pseudohomophone advantage from parafoveal preview. Collapsed across orthographic similarity, the magnitude of the pseudohomophone advantage was: 9 ms (first fixation) and 16 ms (gaze) for the TDO group; 12 ms (first fixation) and 16 ms (gaze) for the DO group; 7 ms (first fixation) 40 ms (gaze) for the TDY group; 26 ms (first fixation) and -3 ms (gaze) for the DY group. The pattern of effects involving the younger group with a history of dyslexia seems somewhat noisy, and it seems likely that analyses involving this participant group were undermined by low statistical power, as there were only 14 younger teenagers with a history of dyslexia but were over 20 teenagers in each of the other three groups. Again, we calculated Bayes factor to compare two models, one where group interacted with the phonological manipulation (group*phoncond) against one where the interaction was not included (group+phoncond), as per Experiment 1. The Bayes factor was 0.025 for first fixation duration, and 0.032 for gaze duration, showing that the model without the interaction between group and the phonological manipulation was the better fit to the data. This result is consistent with the non-significant interaction terms in the main LME models.

There was also an effect of the orthographic manipulation, whereby previews that were orthographically similar to the correctly spelled base word resulted in faster reading times than previews that were orthographically dissimilar (single fixation duration = 41ms difference; first fixation duration = 32ms difference; gaze duration = 308ms difference) (Term 5 in Table 8). Finally, there were two significant three-way interactions, between the two experimental manipulations and the comparison of older teenagers with a history of dyslexia to both typically developing control groups, that was significant for first and single fixation durations (Terms 13 and 14 in Table 8). As can be seen in Table 6, the cost associated with nonwords that were orthographically dissimilar to their correctly spelled base word was substantially greater when the older teenagers with a history of dyslexia received a spelling control preview (83 ms cost) than when they received a pseudohomophone preview (22 ms cost), or when the older typically developing teenagers received either type of nonword preview (10 ms and 38 ms costs, respectively).

## Discussion

As in Experiment 1, we found a pseudohomophone advantage for all four participant groups. Here, the effect was found in parafoveal pre-processing such that a preview that was phonologically consistent with the correct target word resulted in shorter reading times once that target word was directly fixated than a preview that was phonologically inconsistent. This suggests that all participant groups were able to engage in phonological recoding during parafoveal pre-processing. Note that the numerical means, when collapsed across orthographic similarity, indicated that there was no clear pseudohomophone advantage within the data from the younger teenagers with a history of dyslexia. Such a pattern (an overall main effect of the phonological manipulation, but with numerical differences in the condition means only occurring for three of the four participant groups) would be expected to result in a significant interaction between that participant group and the phonological manipulation but, in our analyses, the effect did not approach significance (and this was supported by our Bayes factor calculation). There is clearly no statistical evidence for an interaction with participant group; however, given the smaller sample size for younger teenagers with a history of dyslexia, we are reluctant to interpret the pseudohomophone advantage in this group. We restrict our discussion and conclusions on this point, therefore, to the older teenagers with and without a history of dyslexia, as well as the younger typically developing teenagers.

Parafoveal pre-processing of phonology was predicted for the older typically developing teenagers, but not for the older teenagers with a history of dyslexia. No previous studies, to date, have examined parafoveal pre-processing of phonology during silent reading in individuals with dyslexia. The most relevant data to the present study were reported by Chace *et al*. (2005) who found that less skilled adult readers demonstrated no evidence of parafoveal pre-processing of phonology during reading [35]. It was, therefore, somewhat surprising that the teenagers with a history of dyslexia in the present study showed a pseudohomophone advantage from parafoveal preview. There are two likely reasons for this apparent discrepancy between the two studies. First, the properties of the pretarget word in the sentences. Chace *et al*. used pretarget words that were five to eight letters long, typically adjectives, with a mean frequency of 10,775 counts per million. Whilst not specifically mentioned, the probable reason for Chace *et al*.’s choice of these controls would have been to minimise skipping of the pretarget word. In the present study, pretarget words were typically both shorter (mean = 4 letters) and of higher frequency (mean = 13,616 counts per million) than those used by Chace *et al*. Whilst the absolute values for the frequency counts cannot be directly compared, as different databases were used to generate them, the indication of an overall difference is unsurprising given our intention of writing sentences that were suitable for readers with dyslexia (in contrast to Chace *et al*.’s stimuli that were written for university students). It is clear that such differences in the pretarget words could well affect pre-processing of the target words, with a number of studies showing that greater foveal processing difficulty reduces parafoveal pre-processing [72,73]. Chace *et al*. concluded that, within their sample of adults, poor readers were required to allocate greater processing resources to the pretarget word and, therefore, were reduced in their parafoveal pre-processing of the target word. In the present study, it may be the case that participants, including those with a history of dyslexia, were more easily able to identify the pretarget word and so greater pre-processing of the target word occurred. The second likely reason is the semantic constraint of the sentence frames. In the present study, the sentences were pre-screened in order to ensure that they were semantically constraining to the target words, with a minimum probability of 0.60). It has previously been demonstrated that parafoveal pre-processing may be facilitated for words that are highly constrained within the sentence context [74]. Chace *et al*. report target word predictabilities of 0.25, and so it seems likely that the reduced semantic constraint of the sentence frames for the target words in that study may also have reduced the extent to which their participants were able to parafoveally pre-process the target words as compared with the situation in the current experiment.

Finally, as predicted, all participant groups showed faster reading times after previews that were orthographically similar to the target word than previews that were orthographically dissimilar. This supports previous research with typically developing beginning readers [65], showing sensitivity to orthography in the parafovea from a relatively young age, and with relatively low reading skills compared to the skilled adult readers who typically comprise the sample for such research. The data also showed that the teenagers with a history of dyslexia were particularly disrupted by orthographically dissimilar, spelling control previews. This was consistent with the data from Experiment 1, and is considered more fully in the General Discussion.

## General discussion

First and foremost, the data demonstrate that teenagers with a history of dyslexia engage in phonological recoding (the subconscious activation of abstract phonological codes from orthography) as part of lexical identification during silent sentence reading, both during foveal processing and parafoveal pre-processing. Whilst the characteristics of the stimuli used seem likely to have facilitated parafoveal pre-processing in Experiment 2, it remains clear that the pseudohomophone advantage must be attributable to phonological recoding. Critically, any faster reading times on pseudohomophones, relative to spelling controls, can only be attributable to activation of phonological codes, given the extensive matching of the two types of nonwords on other variables.

Our observation of phonological recoding in teenagers with a history of dyslexia was surprising, given the well-documented phonological deficits that are thought to play a causal role in dyslexia, and we note that, using a traditional, pen and paper assessment of pseudoword decoding, we did show the expected deficit within our sample of participants with a history of dyslexia. Thus, a comparison the different types of data collected for these experiments makes very clear the differential sensitivity of the tasks used. The pseudoword decoding task, that is often used as part of an assessment of reading and related abilities, showed that the teenagers with a history of dyslexia had a significant deficit compared to their typically developing peers. In contrast, eye movement recordings are a detailed and sensitive index of cognitive processing and, here, provide clear evidence of phonological recoding in lexical identification during silent sentence reading in teenagers with a history of dyslexia that does not differ to that observed in their typically developing peers.

Recall that there are two key differences between these two types of task, which may explain the difference in sensitivity to detecting phonological processing: (1) the pseudoword decoding task uses nonword stimuli that do not map onto any real word lexical entries, whereas the stimuli used in both the eye movement experiment and those from the elision and blending tasks do map onto real word lexical entries; and (2) in the eye movement experiment, further support for processing of the target nonwords was provided by the semantic and syntactic context of the sentences, but no such support is available in an isolated nonword task such as pseudoword decoding. It is clear, therefore, that both the ecological validity of sentence reading tasks, and the sensitivity of eye movement recordings to cognitive processing, make such data ideally suited for studying reading. In the present experiment, those data indicated that teenagers with a history of dyslexia engage in phonological recoding during reading, in a comparable manner to their typically developing peers. There are two possible caveats to this interpretation. First, the increased reliance on top-down processing that has been observed in individuals with dyslexia, possibly increasing their prediction of upcoming words in a sentence [60]. Thus, it may have been the case that individuals with a history of dyslexia were able to predict the identity of the target word from the preceding sentence frame, and so reduce their identification times for that word, more than their typically developing peers did. Second, the target words in the present stimulus set were relatively short and high frequency, to avoid presenting readers with a history of dyslexia with words that they did not know. It might be the case that phonological recoding was possible for these words, but would not be observed when teenagers with a history of dyslexia read longer, or lower frequency words. Numerical trends within the data from the present studies did provide some indication that the pseudohomophone advantage was reduced in the younger teenagers with a history of dyslexia but, although interesting, this aspect of the data was not conclusive. In order to understand these results further, a future examination of the nature of the developmental trajectory of phonological recoding associated with dyslexia will be vital.

When interpreting these data, it is important to note that the participants with a history of dyslexia in these experiments had relatively good reading abilities, given their diagnosis. Three points should be considered here. First, their age—dyslexia has been extensively studied in earlier childhood, but far less so within teenaged populations. It is possible that many individuals with dyslexia who are diagnosed and then benefit from specialised intervention programmes as part of their education might learn strategies and skills that allow them to substantially improve their measured reading performance. Although some work does indicate that reading difficulties in dyslexia persist into adulthood [60,75], the second point may also contribute here—the degree to which particular individuals are able to compensate for their reading difficulties over time. These participants were at a well-performing school in a relatively affluent area, and had attended that school for between two and seven years. Thus, it is reasonable to infer that these individuals were benefitting from strong educational opportunities and individual support, which may well have allowed them to compensate for their diagnosed reading difficulties. Third, we matched our samples of teenagers with and without a history of dyslexia on word reading accuracy, which is only one of a number of aspects of an individual’s reading ability (e.g., accuracy, fluency, and comprehension). Our analyses and conclusions are based upon eye movement measures that reflect lexical identification during reading, relating to our experimental manipulations of carefully controlled target words. The issues surrounding alternative group matching procedures have been explored within the literature [76,77,78,79]. Such issues require investigation, beyond the scope of the present experiments, but may limit the extent to which these data generalise to the wider population of individuals with dyslexia.

We did observe differences in reading behaviour between the teenagers with and without a history of dyslexia in relation to our manipulations of orthographic similarity. In Experiment 1, we found that the older teenagers with a history of dyslexia showed a greater cost to reading from nonwords that were orthographically dissimilar to their base words, relative to their typically developing peers. In Experiment 2, we found a similar effect that was more specifically tied to the spelling controls–those words that were not matched to their correctly spelled base words for either phonology or orthography, and that had been deliberately manipulated in order to have a relatively small orthographic overlap (e.g., *bewl* as a spelling control for *ball/borl*). These nonword stimuli were particularly disruptive for the teenagers with a history of dyslexia, despite the fact that the sentences were highly semantically constraining towards the identity of the correctly spelled base word. One possibility is that, earlier in literacy development, children with dyslexia are more reliant on orthographic information for lexical identification given their phonological processing deficits. Over time, and with accumulating reading experience and ongoing formal literacy instruction (often including interventions that are specific to phonological processing), these children may well reduce or even eliminate their phonological processing difficulties. Such a developmental change could result in those readers engaging in phonological recoding during reading whilst maintaining their longer-term reliance on the orthography of the printed words. Whilst highly speculative, such developmental changes could well result in the pattern of results observed in the present study; again, similar research with younger children with dyslexia, ideally using a longitudinal design, would be necessary in order to evaluate this hypothesis.

To conclude, when compared against both chronological age-matched and word reading accuracy-matched typically developing control groups, our data do not indicate either delayed development of, or atypical, phonological recoding as part of lexical identification in teenagers with a history of dyslexia. Further research is necessary, though, to examine whether such patterns might be modulated by the characteristics of the stimuli used. In contrast, we did find evidence that the teenagers with a history of dyslexia were more dependent upon orthographic form for lexical processing. Further research with younger readers with a history of dyslexia, using natural sentence reading paradigms, will be necessary in order to understand how these individuals develop their processing of different features of printed words as they progress from single word identification to being fluent sentence readers.

## References

[1] Frost R. (1998). Toward a strong phonological theory of visual word recognition: true issues and false trails. *Psychological Bulletin*, 123, 71–99. 10.1037/0033-2909.123.1.71 9461854

[2] Rayner K., Pollatsek A., & Binder K. S. (1998). Phonological codes and eye movements in reading. *Journal of Experimental Psychology*: *Learning*, *Memory*, *and Cognition*, 24, 476–497. 10.1037//0278-7393.24.2.476 9530845

[3] Snowling M.J. (2000). *Dyslexia*. Blackwell: Oxford.

[4] Liberman I. Y. (1973). 1. Segmentation of the spoken word and reading acquisition. *Annals of Dyslexia*, 23, 64–77.

[5] Vellutino, F. R. (1979). Dyslexia: Theory and research. MIT Press.

[6] Vellutino F.R., Fletcher J.M., Snowling M.J., & Scanlon D.M. (2004). Specific reading disability (dyslexia): What have we learned in the past four decades? *Journal of Child Psychology and Psychiatry*, 45, 2–40. 10.1046/j.0021-9630.2003.00305.x 14959801

[7] Snowling M. J. (1981). Phonemic deficits in developmental dyslexia. *Psychological Research*, 43, 219–234. 10.1007/bf00309831 7302091

[8] Stanovich K. E. (1988). Explaining the differences between the dyslexic and the garden-variety poor reader: The phonological-core variable-difference model. *Journal of Learning Disabilities*, 21, 590–604. 10.1177/002221948802101003 2465364

[9] Ramus F., Rosen S., Dakin S. C., Day B. L., Castellote J. M., White S., et al (2003). Theories of developmental dyslexia: insights from a multiple case study of dyslexic adults. *Brain*, 126, 841–865. 10.1093/brain/awg076 12615643

[10] Bradley L., & Bryant P. E. (1978). Difficulties in auditory organisation as a possible cause of reading backwardness. *Nature*, 271, 746–747. 10.1038/271746a0 625341

[11] Bradley L., & Bryant P. E. (1983). Categorizing sounds and learning to read–A casual connection. *Nature*, 301, 419–421.

[12] Elbro C., & Jensen M. N. (2005). Quality of phonological representations, verbal learning, and phoneme awareness in dyslexic and normal readers. *Scandinavian Journal of Psychology*, 46, 375–384. 10.1111/j.1467-9450.2005.00468.x 16014082

[13] Szenkovits G., & Ramus F. (2005). Exploring dyslexics’ phonological deficit I: lexical vs. sub-lexical and input vs. output processes. *Dyslexia*, 11, 253–268. 10.1002/dys.308 16355747

[14] Goswami U. (2011). A temporal sampling framework for developmental dyslexia. *Trends in Cognitive Sciences*, 15, 3–10. 10.1016/j.tics.2010.10.001 21093350

[15] Nicolson R. I., & Fawcett A. J. (1990). Automaticity: A new framework for dyslexia research? *Cognition*, 35, 159–182. 10.1016/0010-0277(90)90013-a 2354611

[16] Stein J. (2001). The magnocellular theory of developmental dyslexia. *Dyslexia*, 7, 12–36. 10.1002/dys.186 11305228

[17] O’Brien B.A., van Orden G.C., & Pennington B.F. (2013). Do dyslexics misread a ROWS for a ROSE? *Reading and Writing*, 26, 381–402. 10.1007/s11145-012-9373-8 24791075 PMC4004072

[18] Ramus F., & Szenkovits G. (2008). What phonological deficit? *The Quarterly Journal of Experimental Psychology*, 61, 129–141. 10.1080/17470210701508822 18038344

[19] Ramus F., & Ahissar M. (2012). Developmental dyslexia: The difficulties of interpreting poor performance, and the importance of normal performance. *Cognitive Neuropsychology*, 29, 104–122. 10.1080/02643294.2012.677420 22559749

[20] Kirkby J.A., Webster L.A.D., Blythe H.I., & Liversedge S.P. (2008). Binocular coordination during reading and non-reading tasks. *Psychological Bulletin*, 134, 742–763. 10.1037/a0012979 18729571

[21] Rayner K. (1985a). Do faulty eye movements cause dyslexia? *Developmental Neuropsychology*, 1, 3–15.

[22] Rayner K. (1985b). The role of eye movements in learning to read and reading disability. *Remedial and Special Education*, 6, 53–60.

[23] Blythe H.I., Kirkby J.A., & Liversedge S.P. (2018). Comments on: "What is Developmental Dyslexia?" *Brain Sci*. 2018, 8, 26 The Relationship between Eye Movements and Reading Difficulties. *Brain Sciences*, *8*, 100. 10.3390/brainsci8060100 PMC6025640 29867069

[24] Hutzler F., Kronbichler M., Jacobs A. M., & Wimmer H. (2006). Perhaps correlational but not causal: No effect of dyslexic readers’ magnocellular system on their eye movements during reading. *Neuropsychologia*, 44, 637–648. 10.1016/j.neuropsychologia.2005.06.006 16115655

[25] Bauer D. W., & Stanovich K. E. (1980). Lexical access and the spelling-to-sound regularity effect. *Memory &* *Cognition*, 8, 424–432. 10.3758/bf03211139 7442545

[26] Lesch M. F., & Pollatsek A. (1993). Automatic access of semantic information by phonological codes in visual word recognition. *Journal of Experimental Psychology*: *Learning*, *Memory*, *and Cognition*, 19, 285–294. 10.1037//0278-7393.19.2.285 8454962

[27] Seidenberg M. S., Waters G. S., Barnes M. A., & Tanenhaus M. K. (1984). When does irregular spelling or pronunciation influence word recognition? *Journal of Verbal Learning and Verbal Behavior*, 23, 383–404.

[28] Coltheart M., Davelaar E., Jonasson T., & Besner D. (1977). Access to the internal lexicon In Dornic S. (Ed.), *Attention and Performance VI* (pp. 535–555). Hillsdale, NJ: Erlbaum.

[29] McCann R. S., Besner D., & Davelaar E. (1988). Word recognition and identification: Do word-frequency effects reflect lexical access? *Journal of Experimental Psychology*: *Human Perception and Performance*, 14, 693–706.

[30] Meyer D. E., Schvaneveldt R. W., & Ruddy M. G. (1974). Functions of graphemic and phonemic codes in visual word-recognition. *Memory & Cognition*, 2, 309–321. 24214761 10.3758/BF03209002

[31] Rubenstein H., Lewis S. S., & Rubenstein M. A. (1971). Evidence for phonemic recoding in visual word recognition. *Journal of Verbal Learning and Verbal Behavior*, 10, 645–657.

[32] van Orden G. C. (1987). A ROWS is a ROSE: Spelling, sound, and reading. *Memory & Cognition*, 15, 181–198. 3600258 10.3758/bf03197716

[33] van Orden G. C., Johnston J. C., & Hale B. L. (1988). Word identification in reading proceeds from spelling to sound to meaning. *Journal of Experimental Psychology*: *Learning*, *Memory*, *and Cognition*, 14, 371–386. 10.1037//0278-7393.14.3.371 2969938

[34] Leinenger M. (2014). Phonological coding during reading. *Psychological Bulletin*, 140, 1534–1555. 10.1037/a0037830 25150679 PMC4211933

[35] Chace K.H., Rayner K., & Well A.D. (2005). Eye movements and phonological parafoveal preview: Effects of reading skill. *Canadian Journal of Experimental Psychology*, 59, 209–217. 10.1037/h0087476 16248500

[36] Miellet S., & Sparrow L. (2004). Phonological codes are assembled before word fixation: Evidence from boundary paradigm in sentence reading. *Brain and Language*, 90, 299–310. 10.1016/S0093-934X(03)00442-5 15172547

[37] Pollatsek A., Lesch M. Morris R.K., & Rayner K. (1992). Phonological codes are used in integrating information across saccades in word identification and reading. *Journal of Experimental Psychology*: *Human Perception and Performance*, 18, 148–162. 10.1037//0096-1523.18.1.148 1532185

[38] Blythe H.I., Pagan A., & Dodd M. (2015). Beyond decoding: phonological processing during silent reading in beginning readers. *Journal of Experimental Psychology*: *Learning*, *Memory*, *and Cognition*, 41, 1244–1252. 10.1037/xlm0000080 25528096

[39] Jared D., Ashby J., Agauas S.J., & Levy B.A. (2015). Phonological activation of word meanings in Grade 5. *Journal of Experimental Psychology*: *Learning*, *Memory*, *and Cognition*, 42, 524–541. 10.1037/xlm0000184 26436634

[40] Lukatela G., & Turvey M. T. (1994). Visual lexical access is initially phonological: I. Evidence from associative priming by words, homophones, and pseudohomophones. *Journal of Experimental Psychology*: *General*, 123, 107–128. 8014609 10.1037//0096-3445.123.2.107

[41] Olson R. K., Kliegl R., Davidson B.J., & Foltz G. Individual and developmental differences in reading disability In MacKinnon G.E. and Waller T.G. (Eds.), Reading research: Advances in theory and practice, Vol. 4 New York: Academic Press, 1985 pp. 1–64.

[42] Bergmann J., & Wimmer H. (2008). A dual-route perspective on poor reading in a regular orthography: Evidence from phonological and orthographic lexical decisions. *Cognitive Neuropsychology*, 25, 653–676. 10.1080/02643290802221404 18642138 PMC2976852

[43] Hawelka S., Gagl B., & Wimmer H. (2010). A dual-route perspective on eye movements of dyslexic readers. *Cognition*, 115, 367–379. 10.1016/j.cognition.2009.11.004 20227686 PMC2976468

[44] Wagner R.K., & Torgesen J.K. (1987). The nature of phonological processing and its causal role in the acquisition of reading skills. *Psychological Bulletin*, 101, 192–212.

[45] Wagner R.K., Torgesen J.K., & Rashotte C.A. (1994). Development of reading-related phonological processing abilities: New evidence of bidirectional causality from a latent variable longitudinal study. *Developmental Psychology*, 30, 73–87.

[46] Wechsler (2005). Wechsler Individual Achievement Test (WIAT-II UK). Harcourt Assessment.

[47] Wagner, R. K., Torgesen, J. K., & Rashotte, C. A. (1999). CTOPP: Comprehensive test of phonological processing. Pro-ed.

[48] Dunn, L. M., & Dunn, D. M. (2009). The British Picture Vocabulary Scale. GL Assessment Ltd.

[49] Raven, J. C., Court, J.H., & Raven, J. (1998). Progressive matrices standard. Pearson Education Ltd.

[50] Balota D.A., Yap M.J., Cortese M.J., Hutchison K.A., Kessler B., Loftis B., et al (2007). The English Lexicon Project. *Behavior Research Methods*, 39, 445–459. 10.3758/bf03193014 17958156

[51] Masterson, J., Stuart, M., Dixon, M., Lovejoy, D., & Lovejoy, S. (2003). The children’s printed word database. 10.1348/000712608X371744 20021708

[52] Kuperman V., Stadthagen-Gonzalez H., & Brysbaert M. (2012). Age-of-acquisition ratings for 30,000 English words. *Behavior Research Methods*, 44, 978–990. 10.3758/s13428-012-0210-4 22581493

[53] Blythe H.I., Dickins J.H., Kennedy C.R., & Liversedge S.P. (2018). Phonological processing during silent reading in teenagers who are deaf/hard of hearing: An eye movement investigation. *Developmental Science*, e12643 10.1111/desc.12643 29356239

[54] Bates, D., Maechler, M., & Bolker, B. (2014). lme4: Linear mixed-effects models using S4 classes. (R package version 0.999999–0). http://CRAN.project.org/package=lme4.

[55] R Core Team. R (2014). A language and environment for statistical computing, R foundation for statistical computing Vienna, Austria http://www.R-project.org/

[56] Baayen R. H., Davidson D. J. & Bates D. M. (2008). Mixed-effects modelling with crossed random effects for subjects and items. *Journal of Memory and Language*, 59, 390–412.

[57] Kass R.E. & Raftery A.E. (1995). Bayes factors. *Journal of the American Statistical Association*, 90, 773–795.

[58] Rouder J. N., Morey R. D., Speckman P. L., & Province J. M. (2012). Default Bayes factors for ANOVA designs. *Journal of Mathematical Psychology*, 56, 356–374.

[59] Morey, R.D., & Rouder, J.N. (2015). BayesFactor: Computation of Bayes factors for common designs. R package version 0.9.12–2. https://CRAN.R-project.org/package=BayesFactor

[60] Bruck M. (1990). Word-recognition skills of adults with childhood diagnoses of dyslexia. *Developmental Psychology*, 26, 439–454.

[61] Rayner K., Liversedge S. P., & White S. J. (2006). Eye movements when reading disappearing text: The importance of the word to the right of fixation. *Vision Research*, 46, 310–323. 10.1016/j.visres.2005.06.018 16085229

[62] Hyönä J. (2011). Foveal and parafoveal processing during reading In Liversedge S., Gilchrist I., and Everling S. (Eds.) *Oxford Handbook of Eye Movements* (pp. 819–838). Oxford, UK: Oxford University Press.

[63] Häikiö T., Bertram R., & Hyönä J. (2010). Development of parafoveal processing within and across words in reading: Evidence from the boundary paradigm. *The Quarterly Journal of Experimental Psychology*: *Human Experimental Psychology*, 63, 1982–1998. 10.1080/17470211003592613 20336584

[64] Marx C., Hawelka S., Schuster S., & Hutzler F. (2015). An incremental boundary study on parafoveal preprocessing in children reading aloud: Parafoveal masks overestimate the preview benefit. *Journal of Cognitive Psychology*, 27, 549–561. 10.1080/20445911.2015.1008494 26246890 PMC4487581

[65] Pagan A., Blythe H.I., & Liversedge S.P. (2016). Parafoveal pre-processing of word initial trigrams during reading in adults and children. *Journal of Experimental Psychology*: *Learning*, *Memory*, *and Cognition*, 42, 411–432. 10.1037/xlm0000175 26348198

[66] Tiffin-Richards S. P., & Schroeder S. (2015). Children’s and adults’ parafoveal processes in German: Phonological and orthographic effects. *Journal of Cognitive Psychology*, 27, 531–548.

[67] Binder K. S., Pollatsek A. & Rayner K. (1999). Extraction of information to the left of the fixated word in reading. *Journal of Experimental Psychology*: *Human Perception and Performance*, 25, 1162–1172. 10464948

[68] Johnson R. L., & Dunne M. D. (2012). Parafoveal processing of transposed-letter words and nonwords: Evidence against parafoveal lexical activation. *Journal of Experimental Psychology*: *Human Perception and Performance*, 38, 191–212. 10.1037/a0025983 22060141

[69] McConkie G. W., & Zola D. (1979). Is visual information integrated across successive fixations in reading? *Perception & Psychophysics*, 25, 221–224. 461078 10.3758/bf03202990

[70] Rayner K., McConkie G. W., & Zola D. (1980). Integrating information across eye movements. *Cognitive Psychology*, 12, 206–226. 10.1016/0010-0285(80)90009-2 7371377

[71] Rayner K. (1975). The perceptual span and peripheral cues in reading. *Cognitive Psychology*, 7, 65–81.

[72] Henderson J. M., & Ferreira F. (1990). Effects of foveal processing difficulty on the perceptual span in reading: implications for attention and eye movement control. *Journal of Experimental Psychology*: *Learning*, *Memory*, *and Cognition*, 16, 417–429. 10.1037//0278-7393.16.3.417 2140401

[73] White S. J., Rayner K., & Liversedge S. P. (2005). Eye movements and the modulation of parafoveal processing by foveal processing difficulty: A reexamination. *Psychonomic Bulletin & Review*, 12, 891–896. 16524007 10.3758/bf03196782

[74] Balota D. A., Pollatsek A., & Rayner K. (1985). The interaction of contextual constraints and parafoveal visual information in reading. *Cognitive Psychology*, 17, 364–390. 10.1016/0010-0285(85)90013-1 4053565

[75] Bruck M. (1992). Persistence of dyslexics’ phonological awareness deficits. *Developmental Psychology*, 28, 874–886.

[76] Van den Broeck W., Geudens A., & van den Bos K. P. (2010). The nonword-reading deficit of disabled readers: A developmental interpretation. *Developmental Psychology*, 46, 717–734. 10.1037/a0019038 20438182

[77] Van den Broeck W., & Geudens A. (2012). Old and new ways to study characteristics of reading disability: The case of the nonword-reading deficit. *Cognitive Psychology*, 65, 414–456. 10.1016/j.cogpsych.2012.06.003 22859020

[78] Parrila R., Dudley D., Song S., & Georgiou G. K. (2019). A meta-analysis of reading-level match dyslexia studies in consistent alphabetic orthographies. *Annals of Dyslexia*, 1–26. 31664608 10.1007/s11881-019-00187-5

[79] Parrila R., Georgiou G. K., & Papadopoulos T. C. (2020). Dyslexia in a consistent orthography: Evidence from reading-level match design. *Dyslexia*, 1–16. 10.1002/dys.1650 32011776

